# The Spitting Nuisance

**Published:** 1896-07

**Authors:** 


### Editorial.

### THE SPITTING NUISANCE.

An exchange contains the following item: "A Good Example.—The city of Sidney, Australia," says the *Boston Medical and Surgical Journal* "has imposed a fine of one pound sterling upon any person convicted of spitting upon the floor of public buildings or upon the street."

Such an ordinance might be carried out successfully in the antipodes, but it would prove a galling restriction on this side of the globe. Foreigners justly regard spitting as one of the most conspicuous characteristics of the American. Time and again it has been made a matter of comment by the several Post-Columbian discoverers of America. Dickens, some forty years ago, in his "American Notes," made much ill-humored mention of this objectionable Americanism.

Some of the most noted Americans have been chewers, and therefore, spitters. It is said that John Y. Mason, minister plenipotentiary from the Confederate States to the Court of St. James, during his stay in London, built up the reputation of being the most accurate spitter at long range who ever came to the shores of Briton. It was his boast that he could bury a fly at six feet with unfailing accuracy and rapidity, and without even the assistance of two finger-tips pressed against his lips to steady his aim.

Spitting is, of course, world-wide, but indiscriminate spitting is almost exclusively limited to America. The scarcity of spittoons is very noticeable in Europe, and shows plainly that there is but little use for them. The prevalence of the tobacco chewing habit in America is probably at the foundation of the spitting nuisance, but it has long since ceased to be the exciting cause. It merely set the fashion, other factors helped it on until now it is not simply an objectively disagreeable mode of ridding the mouth of the accumulated secretion of the salivary glands, plain or mixed with the expressed juices of the weed nicotian, but it has become an act that is more than mechanical—it can be made the expression of emotions.

A quick side-fling of the head with lateral expectoration is expressive of a light degree of contempt, and is much in vogue among young gentlemen of the Chimmey Fadden persuasion.

Elongation of the neck, retraction of the head with the hand in case the former (and it is apt to be) is of the patri archal type, with long slow expectoration, means deliberation, and is employed to gain time in formulating a reply to an important question.

To spit several times, jerkily and in rapid succession, indicates anger or mental perturbation.

To take off the coat, roll up the sleeves and spit—loudly and emphatically upon the hands, in some parts of the country means "Walk in," in others it means "Get out quickly." A quick and furtive squirt from behind the hand means shyness, reserve, timidity.

The method of spitting through the interdental spaces with the tip of the tongue engaged between the teeth is adopted almost exclusively by negroes, and will be mentioned here only to be condemned.

Explosive spitting with the lips separated and accompanied by a blowing sound indicates inexperience, and warns the bystander to put up an umbrella.

---

The Board of Health of New York City and the medical press are waging war against spitting. This is eminently proper. Restrictive measures might prove of use in preventing the spread of tuberculosis, but let repressive efforts be tempered with discrimination. Don't permit the iconoclastic tendencies of the age to drag down and destroy a time honored custom of the great republic. Let not sumptuary law curtail the right of the great American tobacco-chewer. Now is a convenient time for them to form a protective association, so that by cooperation they may secure the undisturbed enjoyment of all the rights and privileges of a free man. "*Civis Americanus Sum* and can spit where I choose" is a good watchword.

---

### OF INTEREST TO COUNTRY SUBSCRIBERS.

Yielding to the universal protest, the committee of management of the International Medical Congress of Moscow in 1897, have announced that English and German will be accepted as official languages with French and Russian. This will be a great relief to those who were preparing to couch their ideas in the Muscovite tongue.

